# Downregulation of microRNA-100 enhances the ICMT-Rac1 signaling and promotes metastasis of hepatocellular carcinoma cells

**DOI:** 10.18632/oncotarget.2601

**Published:** 2014-10-18

**Authors:** Hui-Chao Zhou, Jian-Hong Fang, Xu Luo, Lei Zhang, Jine Yang, Chong Zhang, Shi-Mei Zhuang

**Affiliations:** ^1^ Key Laboratory of Liver Disease of Guangdong Province, The Third Affiliated Hospital of Sun Yat-sen University, Guangzhou, P. R. China; ^2^ Key Laboratory of Gene Engineering of the Ministry of Education, State Key Laboratory of Biocontrol, School of Life Sciences, Sun Yat-sen University, Guangzhou, P. R. China

**Keywords:** miR-100, non-coding RNA, Rac1, ICMT, hepatocellular carcinoma

## Abstract

Metastasis is responsible for rapid recurrence of hepatocellular carcinoma (HCC) and poor survival of HCC patients. Here we showed that miR-100 downregulation in HCC tissues was significantly associated with venous invasion, advanced TNM stage, tumor nodule without complete capsule, poorer cell differentiation, and shorter recurrence-free survival. Both gain- and loss-of-function studies showed that miR-100 dramatically suppressed the ability of HCC cells to migrate and to invade through Matrigel *in vitro*. Analyses using mouse orthotopic xenograft model further revealed that xenografts of miR-100-stable-expressing HCC cells displayed a significant reduction in pulmonary metastasis, compared with control group. Subsequent investigations revealed that miR-100 directly inhibited the expression of isoprenylcysteine carboxyl methyltransferase (ICMT) and ras-related C3 botulinum toxin substrate 1 (Rac1) by binding to their 3′-UTRs, and in turn suppressed lamellipodia formation and matrix metallopeptidase 2 (MMP2) activation. Furthermore, knockdown of ICMT and Rac1 phenocopied the anti-metastasis effect of miR-100, whereas overexpression of the constitutively active Rac1 (Q61L) antagonized the function of miR-100. Taken together, miR-100 represses metastasis of HCC cells by abrogating the ICMT-Rac1 signaling. Downregulation of miR-100 contributes to HCC metastasis and the restoration of miR-100 is a potential strategy for cancer therapy.

## INTRODUCTION

Hepatocellular carcinoma (HCC) is a common and highly lethal malignancy with increasing incidence globally. It is characterized by high postsurgical recurrence and extremely poor survival, which is attributed to the early frequent metastasis [1]. Therefore, identifying novel molecules that regulate HCC metastasis will promote the development of anti-metastasis strategies. Recently, the classic categories of oncogenes and tumor suppressor genes have been expanded to include a class of evolutionarily conserved small non-coding RNAs, known as microRNAs (miRNAs). miRNAs regulate a large number of protein-coding genes by base-pairing with 3′-untranslated regions (3′-UTRs) of target mRNAs. More than 60% of human protein-coding genes are predicted to contain miRNA-binding sites [2], endowing miRNAs with the capacity to regulate various biological processes, such as cell differentiation, proliferation, apoptosis, motility, etc. Loss or gain of function of specific miRNAs has been demonstrated to contribute to tumorigenesis and cancer progression [3-5]. Although the causal link between miRNA deregulation and tumor metastasis has been identified, the regulatory role of miRNAs in the metastasis of different cancers is still largely unclear.

MicroRNA-100 (miR-100) is frequently downregulated in multiple malignancies. In recent years, important advances have been made in exploring the regulatory function of miR-100 in cell proliferation and apoptosis. miR-100 inhibits growth of bladder and breast cancer cells [6, 7], promotes apoptosis of acute lymphoblastic leukemia, cervical and lung cancer cells [8-10], and enhances chemotherapeutic sensitivity in ovarian cancer cells [11]. Furthermore, polo-like kinase 1 (PLK1), mechanistic target of rapamycin (mTOR), insulin-like growth factor 1 receptor (IGF1R), FK506 binding protein 51 (FKBP51), insulin-like growth factor 2 (IGF2) and homeobox A1 (HOXA1) have been identified as direct targets of miR-100 [6-11], which mediate the growth-inhibitory or apoptosis-promoting effects of miR-100. To date, only three reports investigate the role of miR-100 in HCC. It has been shown that reduced expression of miR-100 is correlated with higher incidence of lymph node metastasis and poor survival of HCC patients [12] and miR-100 inhibits cell growth and induces apoptosis by targeting PLK1 in HCC cells [12, 13]. We find that miR-100 promotes the autophagy of HCC cells by inhibiting the expression of mTOR and IGF-1R [14]. Clearly, the role of miR-100 in HCC development requires more extensive investigation.

We have previously found that the level of miR-100 was significantly decreased in HCC tissues [14, 15]. In the present study, we further showed that miR-100 displayed more pronounced reduction in the HCC tissues with metastatic potential. The restoration of miR-100 expression significantly repressed the *in vitro* migration and invasion and the *in vivo* metastasis of HCC cells. Subsequent mechanism investigations revealed that miR-100 exerted its anti-metastasis function by directly repressing the expression of ICMT and Rac1, and consequently abrogating the Rac1 signaling. These findings provide a novel mechanistic insight into the role of miR-100 in suppressing metastasis, highlight the significance of miR-100 downregulation in HCC progression and implicate miR-100 as a potential therapeutic target for HCC.

## RESULTS

### miR-100 suppresses metastasis of HCC cells *in vitro* and *in vivo*

**Figure 1:**
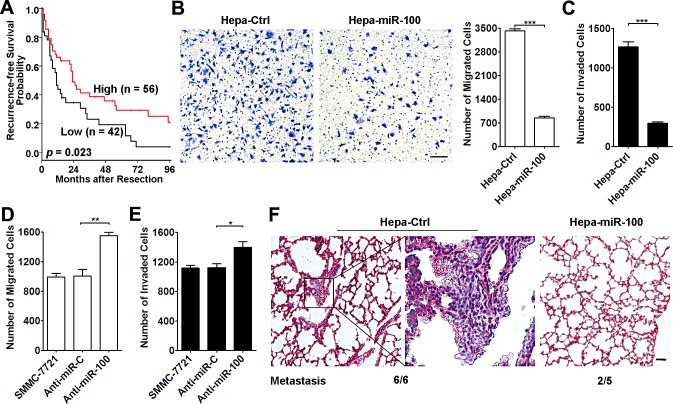
miR-100 inhibits metastasis of HCC cells (A) Lower miR-100 level was associated with shorter recurrence-free survival of HCC patients. Kaplan-Meier survival analysis was performed in 98 HCC patients, who did not get anticancer treatments before tumor relapse. The levels of mature miR-100 were analyzed using qPCR, and the minimum *P*-value approach was used to choose the cutoff point for separating the miR-100-low expression and miR-100-high expression groups. *P* values were calculated by log-rank test. (B and C) Overexpression of miR-100 suppressed the *in vitro* migration and invasion of hepatoma cells. Hepa1-6 cells with stable expression of miR-100 (Hepa-miR-100) and control cells (Hepa-Ctrl) were added to transwell chamber without (B, 5×10^4^) or with (C, 1×10^5^) Matrigel coating. (D and E) Antagonism of miR-100 promoted the *in vitro* migration and invasion of HCC cells. SMMC-7721 cells transfected with anti-miR-C or anti-miR-100 were added to transwell chamber without (D, 5×10^4^) or with (E, 7.5×10^4^) Matrigel coating. (F) Restoration of miR-100 expression inhibited the *in vivo* metastasis of hepatoma cells. Hepa-Ctrl and Hepa-miR-100 cells (1×10^6^) were inoculated under the capsules of the left hepatic lobes of C57BL/6J mice and maintained for 35 days. Representative H&E-stained sections of lung tissues in Hepa-Ctrl and Hepa-miR-100 groups are shown. The number of mice displaying pulmonary metastasis relative to the total number of tumor-bearing mice in each group is indicated in the bottom. Scale bar, 50 μm. *P < 0.05, **P < 0.01, ***P < 0.001.

To explore the significance of miR-100 downregulation in HCC development, the correlation between miR-100 level and the clinical features of HCC patients was first analyzed. The decreased miR-100 expression was significantly associated with venous invasion, advanced TNM stage, tumor nodule without complete capsule and poorer cell differentiation (Table 1). Furthermore, the Kaplan-Meier plots revealed that reduced miR-100 expression was related to shorter recurrence-free survival of HCC patients (Figure 1A). These data suggest that miR-100 downregulation may contribute to HCC metastasis.

**Table 1:** Association of miR-100 Expression with Clinical Features^a^

Variables	Case Number	*P*^b^		Variables	Case Number	*P*
Gender				Tumor size (cm)		
Male	100	0.885		>5	87	0.661
Female	18			≤5	31	
Age-yr				Tumor number		
>50	53	0.106		>1	29	0.157
≤50	65			1	89	
HBV				Tumor capsule		
Positive	102	0.305		None/incomplete	70	**0.016**
Negative	16			Complete	47	
Cirrhosis				No data	1	
Yes	102	0.395		Portal vein tumor thrombus		
No	16			Yes	18	0.392
Ascites				No	98	
Yes	14	0.475		No data	2	
No	104			Venous invasion		
AFP (ng/ml)				Yes	60	**0.026**
≥400	56	**0.003**		No	58	
<400	62			TNM stage		
ALT (U/L)				II/III	51	**0.028**
≥50	54	0.364		I	67	
<50	64			Edmondson grade		
				>II	64	**0.008**
				I-II	54	

a Analysis was conducted on 118 cases. The mature miR-100 level was examined by qPCR and normalized to RNU6B level.

b Association between the miR-100 level and clinical characteristics of patients was analyzed by using unpaired Student *t* test.

To further elucidate the role of miR-100 in HCC metastasis, the miR-100 stable-expressing cell lines were established using mouse Hepa1-6 and human QGY-7703 cells (Supplementary Figure 1 and 2A). The transwell assays were then applied to analyze the effect of miR-100 on *in vitro* migration and invasion of HCC cells. Stable expression of miR-100 in Hepa1-6 (Hepa-miR-100) led to substantial reduction in the number of cells that migrated and invaded through the transwell chamber (Figure 1B and C). Consistently, QGY-miR-100 displayed significantly decreased migration and invasion activity, compared with the control cells (QGY-Ctrl, Supplementary Figure 2B-C). Furthermore, wound healing scratch test revealed that introduction of miR-100 also suppressed the mobility of HCC cells in a two dimensional way (Supplementary Figure 2D). To verify the above findings from gain-of-function studies, loss-of-function analysis was carried out in human SMMC-7721 cells, which showed higher miR-100 level than QGY-7703 and Hepa1-6 cells (Supplementary Figure 3). As shown, suppression of endogenous miR-100 by anti-miR-100 enhanced both the migratory and invasive ability of SMMC-7721 cells (Figure 1D and E).

In order to validate whether miR-100 could inhibit metastasis of HCC cells *in vivo*, Hepa-Ctrl (control) and Hepa-miR-100 cells were implanted into murine livers. In agreement with the *in vitro* results, tumor xenografts generated from Hepa-miR-100 cells displayed a reduced incidence of pulmonary metastasis, compared with Hepa-Ctrl-xenografts (Hepa-Ctrl vs. Hepa-miR-100 groups: 6/6 vs 2/5, Figure 1F).

Collectively, both *in vitro* and *in vivo* studies indicate that miR-100 is able to repress metastasis of HCC cells and its downregulation may facilitate HCC metastasis.

### miR-100 directly inhibits the expression of Rac1 and ICMT

**Figure 2:**
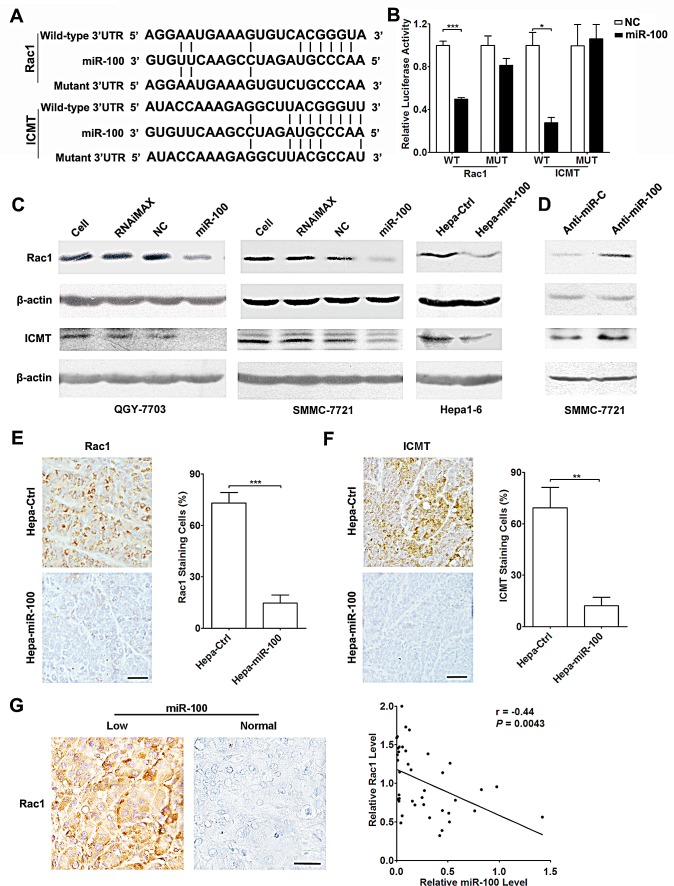
Rac1 and ICMT are direct targets of miR-100 (A) miR-100 and its putative binding sequences in the 3′-UTR of Rac1 and ICMT. Mutations were generated in the complementary site that binds to the seed region of miR-100. (B) miR-100 overexpression suppressed the activity of renilla luciferase that carried the wild-type but not mutant 3′-UTR of Rac1 and ICMT. QGY-7703 cells were co-transfected with the indicated RNA duplex and psiCHECK2 luciferase reporter plasmid containing wild-type or mutant 3′-UTR (indicated as WT or MUT on the X axis) of putative target genes. The values for the luciferase activity assays were from three independent experiments that were performed in duplicate. (C) Reintroduction of miR-100 reduced the endogenous level of Rac1 and ICMT proteins in HCC cell lines. Left and middle panels, QGY-7703 and SMMC-7721 cells without treatment (lane 1), treated with Lipofectamine RNAiMax (lane 2), or transfected with the indicated RNA duplex (lanes 3-4). Right panel, Hepa1-6 stable subclones. (D) Inhibition of miR-100 increased the protein levels of Rac1 and ICMT. Forty-eight hours after transfection with anti-miR-C or anti-miR-100, SMMC-7721 cells were analyzed by immunoblotting. For (C and D), the results were reproducible in three independent experiments. β-actin, internal control. (E and F) Mouse orthotopic xenografts of Hepa-miR-100 cells showed much lower Rac1 and ICMT expression than those of Hepa-Ctrl cells. (G) The level of miR-100 was inversely correlated with Rac1 expression in human HCC tissues. Rac1 expression was quantified based on immunohistochemical staining and miR-100 levels were detected by qPCR. Brown signal was considered as positive staining. Scale bar, 50 μm. *P < 0.05, **P < 0.01, ***P < 0.001.

To identify the downstream molecules responsible for the anti-metastasis function of miR-100, the putative targets of miR-100 were predicted using TargetScan (Release 4.2) and MiRanda databases (August 2010 release). Among them, Rac1 and ICMT were selected for further experimental validation (Supplementary Table 1), because Rac1 is frequently activated in tumor tissues and promotes cancer metastasis [16-18], while ICMT plays an essential role in activating Rho GTPase [19], including Rac1, and inhibition of ICMT leads to decrease of GTP-bound Rac1 [20]. Dual-luciferase reporter analysis revealed that co-transfection of miR-100 significantly suppressed the activity of renilla luciferase with wild-type 3′-UTR of Rac1 or ICMT, whereas this effect was attenuated when the predicted miR-100 binding sites were mutated (Figure 2A and B). Further investigation revealed that reintroduction of miR-100 diminished the endogenous expression of both Rac1 and ICMT proteins (Figure 2C), while inhibition of endogenous miR-100 increased the level of Rac1 and ICMT (Figure 2D). Moreover, the level of miR-100 was negatively related to the expression of ICMT and Rac1 in tumor tissues (Figure 2E-G). These findings indicate that miR-100 may negatively regulate the expression of Rac1 and ICMT by directly binding to their 3′-UTRs.

### miR-100 exerts its anti-metastasis function by abrogating the ICMT-Rac1 signaling

**Figure 3:**
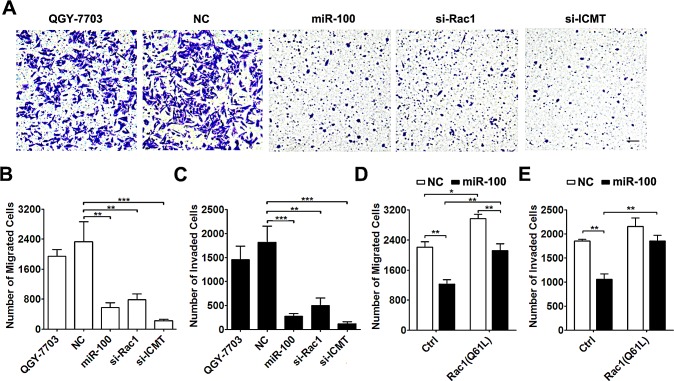
miR-100 exerts its anti-metastasis function by suppressing the ICMT-Rac1 signaling (A-C) Knockdown of either Rac1 or ICMT inhibited the migration and invasion of HCC cells. QGY-7703 cells without transfection or transfected with the indicated RNA duplex were added to transwell chambers without (A and B, 3.5×10^4^) or with (C, 7.5×10^4^) Matrigel coating. Scale bar, 50 μm. **P < 0.01, ***P < 0.001. (D and E) Overexpression of constitutively active Rac1 attenuated the anti-migration and anti-invasion effect of miR-100. QGY-7703 cells that were co-transfected with NC or miR-100 and the Rac1 (Q61L) expression plasmid were submitted to *in vitro* migration (D, 3.5×10^4^) and invasion (E, 7.5×10^4^) assays. *P < 0.05, **P < 0.01.

To evaluate whether ICMT and Rac1 were functional targets of miR-100, siRNA targeting ICMT or Rac1 was transfected into QGY-7703 cells (Supplementary Figure 4). Silencing of either Rac1 or ICMT significantly suppressed *in vitro* migration and invasion of QGY-7703 cells, which phenocopied the effect of miR-100 overexpression (Figure 3A-C). These results were reproducible in another HCC cell line, SMMC-7721 (Supplementary Figure 5). On the other hand, introduction of constitutively active Rac1 (Q61L) [21] into QGY-7703 cells (Supplementary Figure 6) attenuated the suppressive effect of miR-100 on cell migration and invasion (Figure 3D and E).

**Figure 4:**
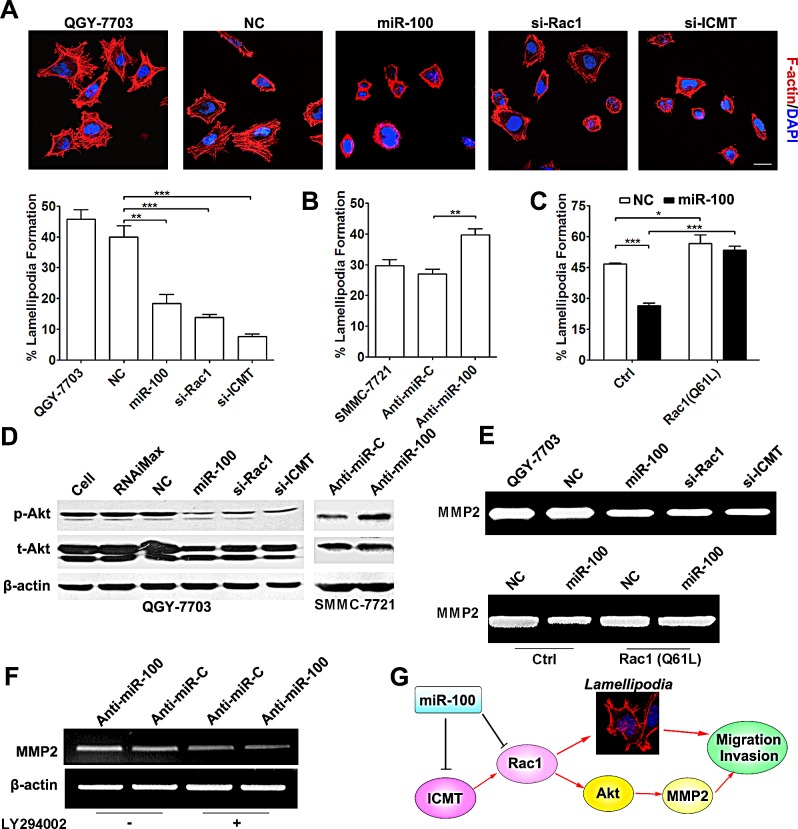
miR-100 inhibits lamellipodia formation and MMP2 activity of HCC cells (A) Restoration of miR-100 expression suppressed lamellipodia formation. QGY-7703 cells without transfection or transfected with the indicated RNA duplex for 48 hours were added to Matrigel-coated plates, incubated for 3 hours, followed by staining for F-actin. Scale bar, 25 μm. **P < 0.01, ***P < 0.001. (B) Antagonism of endogenous miR-100 promoted lamellipodia formation. Forty-eight hours post-transfection, SMMC-7721 cells were analyzed for lamellipodia formation. **P < 0.01. (C) Constitutively active Rac1 abrogated the miR-100-induced suppression of lamellipodia formation. QGY-7703 cells were co-transfected with NC or miR-100 and the Rac1 (Q61L) expression plasmid for 48 hours, then applied to F-actin staining. *P < 0.05, ***P < 0.001. (D) miR-100 attenuated the Akt activity in HCC cells. Forty-eight hours after transfection, the levels of phospho-ser473-Akt (p-Akt) and total Akt (t-Akt) of HCC cells were analyzed by immunoblotting. β-actin, internal control. Left panel, QGY-7703 cells without treatment (lane 1), treated with Lipofectamine RNAiMax (lane 2), or transfected with the indicated RNA duplex (lanes 3-6). Right panel, SMMC-7721 cells transfected with anti-miR-C (lane 1) or anti-miR-100 (lane 2). (E) miR-100 overexpression inhibited MMP2 activity but active Rac1 antagonized this effect. Upper panel, effect of miR-100 and its target genes on MMP2 activity. Lower panel, antagonism of the constitutively active Rac1 (Q61L) against the function of miR-100. Forty-eight hours after transfection, QGY-7703 cells were incubated in serum-free DMEM for 24 hours. TCM was then collected and applied to gelatin zymography analysis. (F) PI3K inhibitor impaired the anti-miR-100-enhanced MMP2 expression. Twenty-four hours after transfection with anti-miR-C or anti-miR-100, SMMC-7721 cells without (−) or with (+) treatment of 10 μM LY294002 for 48 hours were analyzed by RT-PCR. β-actin, internal control. For (D-F), the results were reproducible in three independent experiments and the representative images are shown. (G) The paradigm of miR-100 and its target genes in the regulation of HCC metastasis.

It is reported that Rac1 signaling promotes cell migration by inducing actin polymerization and subsequent lamellipodia formation [22]. Compared with the control groups, miR-100-transfection, similar to the silencing of Rac1 and ICMT, significantly reduced the fraction of cells with lamellipodia (Figure 4A and Supplementary Figure 7A), while the antagonism of miR-100 promoted lamellipodia protrusion (Figure 4B). Additionally, constitutively active Rac1 could abrogate the miR-100-induced suppression of lamellipodia formation (Figure 4C).

It is known that Rac1 can activate PI3K/Akt signaling [23]. Because activation of PI3K/Akt pathway may enhance MMP2 expression [24], which is essential for cell invasion by degrading extracellular matrix, we therefore examined whether the suppression of Rac1 by miR-100 could result in inhibition of Akt and consequently reduced MMP2 activity. As shown, the level of phosphorylated Akt was remarkably reduced in QGY-7703 cells transfected with either miR-100 or siRNA targeting Rac1 or ICMT (Figure 4D, left panel), whereas the phosphorylated Akt was elevated when endogenous miR-100 was antagonized in SMMC-7721 cells (Figure 4D, right panel). Consistently, restoration of miR-100 expression, like knockdown of Rac1 and ICMT, significantly reduced MMP2 activity (Figure 4E, upper panel and Supplementary Figure 7B), while overexpression of constitutively active Rac1 (Q61L) attenuated the inhibitory effect of miR-100 on MMP2 activity (Figure 4E, lower panel). In agreement with the above results, antagonism of endogenous miR-100 by anti-miR-100 led to an increase in MMP-2 level (Figure 4F, lanes 1 and 2), while LY294002, a specific inhibitor of PI3K, abolished the stimulatory effect of anti-miR-100 in MMP2 expression (Figure 4F). These data imply that miR-100 may attenuate MMP2 activity, at least partially, through Rac1-Akt pathway.

Taken together, miR-100 may negatively regulate the ICMT-Rac1 signaling and therefore inhibit metastasis of HCC cells by suppressing lamellipodia formation and MMP2 activation (Figure 4G).

## DISCUSSION

Metastasis is the major cause of tumor recurrence and cancer mortality. miRNAs, a new class of regulators for protein expression, are frequently deregulated in different malignancies. Modulation of miRNA expression has been proposed as an attractive approach for cancer treatments [4, 25]. Downregulation of miR-100 is a prevalent event in various types of cancers. To date, most publications have focused on exploring the regulatory function of miR-100 in cell proliferation and cell death [6-14]. The role of miR-100 in tumor metastasis remained unknown. In the present study, we showed that expression of miR-100 was significantly decreased in HCC with metastatic potential, and both *in vitro* and *in vivo* studies disclosed that the restoration of miR-100 expression dramatically inhibited the metastatic capacity of HCC cells. Mechanism investigations revealed that miR-100 suppressed metastasis of HCC cells by targeting the ICMT-Rac1 signaling. Our data clearly suggest that miR-100 may inhibit the migration, invasion and metastasis of HCC cells and is therefore a potential candidate for anti-metastasis therapy.

Enhanced activity of the Rho GTPases, including Rho, Rac and CDC42, has been implicated in promoting HCC metastasis [26, 27]. Rac1, the most extensively studied isoform in Rac subfamily, is significantly overexpressed in metastatic HCCs and more active in aggressive HCC cell lines [17, 18]. It is also shown that deregulation of the miRNAs that regulate the Rac1 signaling can facilitate tumor cells to acquire a metastatic feature. miR-142-3p, which is downregulated in HCC, can reduce the mRNA and protein level of Rac1, and suppress the migratory and invasive ability of HCC cell lines *in vitro* [28]. We have previously shown that miR-195 downregulation in HCC leads to increased VAV2 expression, which in turn stimulates the Rac1 signaling and thereby promotes metastasis of HCC cells *in vitro* and *in vivo* [29]. Study from He's group reveals that chromosome gain of miR-151 augments metastasis of HCC cells by directly targeting RhoGDIA, and consequently activates Rac1 [30]. In this study, we identified miR-100 as a novel suppressor of the Rac1 signaling and HCC metastasis, based on the following evidences. First, both gain- and loss-of function analyses revealed that miR-100 directly suppressed the expression of Rac1 and its upstream regulator ICMT, and the level of miR-100 was negatively correlated with the level of Rac1 in mouse xenograft and human HCC tissues. Second, inhibition of the ICMT-Rac1 signaling by miR-100 attenuated lamellipodia formation and MMP2 activation, which are the essential events for the migratory and invasive activity of tumor cells. Third, the constitutively active form of Rac1 antagonized the anti-metastatic effect of miR-100 overexpression.

ICMT is an essential post-prenylation-processing enzyme, which methylates a group of proteins including Rho GTPases [19]. Several studies suggest that pharmacological inhibition of ICMT not only induces cell cycle arrest, autophagy and cell death, but also suppresses anchorage-independent colony formation and tumor growth [31, 32]. Moreover, *in vitro* assays indicate that inhibition of ICMT-mediated methylation results in decreased motility of breast tumor cells by reducing the GTP-bound RhoA and Rac1 [20]. Our results suggest that miR-100 simultaneously represses two molecules in the Rac1 signaling pathway, which may provide a more effective prevention for tumor metastasis.

Apart from foremost function on the formation of lamellipodia, Rac1 has been shown to be capable of activating MMP2 [33-35], but the underlying mechanism remains to be elucidated. It is shown that Rac1-enhanced invasion of breast cancer cells could be diminished by the treatment of PI3K inhibitor, LY294002 [36]. However, whether Rac1 activates MMP2 via PI3K/Akt is unknown. In this study, overexpression of miR-100 or knockdown of its target genes Rac1 and ICMT significantly reduced the level of phosphorylated Akt, and inhibition of PI3K/Akt by LY294002 attenuated the anti-miR-100-enhanced MMP2 activity. Moreover, expression of constitutively active Rac1 (Q61L) attenuated the inhibition of miR-100 on MMP2 activity. These data imply that miR-100 may abrogate MMP2 activation, at least partially through suppression of Rac1-Akt signaling transduction.

miR-100 has been reported to inhibit the growth and promote the apoptosis and autophagy of HCC cells [12-14]. In this study, we provide further evidence to support miR-100 as an anti-metastatic miRNA in HCC, which expand our understanding about the regulatory network of miR-100 and the mechanisms of HCC metastasis.

## MATERIALS AND METHODS

### Human tissue specimens

Human HCC tissues were collected from 118 patients who underwent HCC resection at the Cancer Center of Sun Yat-sen University between 2001 and 2006. None of the patients had received any local or systemic anticancer treatments prior to the surgery. The relevant characteristics of the studied subjects are shown in Table 1. Informed consent was obtained from each patient, and the study was approved by the Institute Research Ethics Committee.

### Cell lines

Human HCC cell lines (QGY-7703, SMMC-7721), mouse hepatoma cell line (Hepa1-6) and transformed human embryonic kidney cell line (HEK293T) were maintained in Dulbecco's modified Eagle's medium (DMEM; Life Technologies, MD, USA) that was supplemented with 10% fetal bovine serum (FBS; Hyclone, UT, USA).

The QGY-7703 or Hepa1-6 sublines, which stably expressed miR-100 (QGY-miR-100 or Hepa-miR-100), and the matched control lines (QGY-Ctrl or Hepa-Ctrl) were established using lentiviral expression system as described [14]. Briefly, lentiviruses were generated by transiently co-transfecting HEK293T cells with the lentiviral expression vector (pCDH-miR-100 or pCDH-CMV-MCS-EF1-copGFP, System Biosciences, CA, USA) and packaging plasmid mix (Lenti-X HTX Packaging Mix, Clontech, CA, USA) using calcium phosphate precipitation. Sixteen hours after transfection, cells were refreshed with the complete growth medium and incubated for another 24 hours. The lentiviral supernatants were then harvested and cellular debris was removed by centrifugation at 500 g for 10 minutes. Cells were infected with lentiviruses and the copGFP-expressing cells were sorted by flow cytometry (Beckman Coulter, CA, USA). All sublines were maintained in DMEM with 10% FBS.

### RNA oligoribonucleotides and vectors

All miRNA mimic, small interfering RNA (siRNA) duplexes and miR-100 inhibitor were purchased from GenePharma (Shanghai, China). The small interfering RNA si-Rac1 and si-ICMT targeted the mRNAs that coded for human *Rac1* (NM_006908.4) and *ICMT* (NM_012405.3), respectively. The negative control RNA duplex (NC) for both miRNA mimic and siRNA was non-homologous to any human genome sequence. The inhibitor of miR-100 (anti-miR-100) had a sequence complementary to the mature miR-100. The anti-miR-C, which is non-homologous to any human genome sequences, was used as a negative control for anti-miR-100. Both anti-miR-100 and anti-miR-C were 2′-O-methyl-modified oligoribonucleotides.

To generate the miR-100 lentiviral expression vector (pCDH-miR-100), a 402-bp genomic fragment that encompassed the corresponding miR-100 precursor and its 5′- and 3′-flanking sequences was inserted into the *EcoR*I and *BamH*I sites of pCDH-CMV-MCS-EF1-copGFP (System Biosciences), a lentiviral vector that expresses fluorescent copGFP. The construct was verified by direct sequencing.

Plasmid pcDNA3-EGFP-Rac1 (Q61L) was purchased from Addgene (Plasmid 12981, Cambridge, MA, USA).

To create luciferase reporter constructs psiCHECK2-Rac1-3′UTR-WT and psiCHECK2-ICMT-3′UTR-WT, a wild-type 3′-UTR segments of human *Rac1* (461 bp) or *ICMT* (271 bp) mRNA that contained putative binding sites for miR-100 were inserted downstream the renilla luciferase coding region in psiCHECK2 (Promega, WI, USA). The plasmids psiCHECK2-Rac1-3′UTR-MUT and psiCHECK2-ICMT-3′UTR-MUT, which carried the mutated sequences in the complementary sites of the seed region of miR-100, were produced by site-specific mutagenesis based on psiCHECK2-Rac1-3′UTR-WT and psiCHECK2-ICMT-3′UTR-WT, respectively.

All RNA oligoribonucleotides and primers are provided in Supplementary Table 2.

### Mouse orthotopic xenograft study

All experimental procedures involving animals were performed in accordance with the Guide for the Care and Use of Laboratory Animals (National Institutes of Health publication nos. 80-23, revised 1996) and according to the institutional ethical guidelines for animal experiments. Hepa-Ctrl or Hepa-miR-100 cells (1×10^6^) were resuspended in 25 μl of Matrigel (mixed with 1×PBS at 1:1 volume ratio; cat. 3432-005-01, R&D Systems, MN, USA) and then inoculated under the capsule of the left hepatic lobe of male C57BL/6J mice. Thirty-five days after implantation, the animals were sacrificed. Tumors and lungs were dissected, fixed in formalin and embedded in paraffin. To evaluate the frequency of metastasis, serial sections of lung tissues were stained with hematoxylin-eosin (H&E) and then examined independently by two researchers who were blinded to the treatment.

### Cell transfections

Reverse transfection of RNA oligoribonucleotides was performed using Lipofectamine-RNAiMAX (Life Technologies). A total of 50 nM RNA duplex or 200 nM miRNA inhibitor was used for each transfection. Co-transfection of RNA duplex with plasmid DNA was conducted using Lipofectamine 2000 (Life Technologies). Transfection of HEK293T cells with plasmids was carried out by calcium phosphate precipitation.

### *In vitro* migration and invasion assays

The migration and invasion of tumor cells were analyzed in 24-well Boyden chambers with 8-μm pore size polycarbonate membranes (Corning, NY, USA). For invasion assays, the transwell membranes were coated with Matrigel (R&D Systems) to form matrix barriers. Cells were added to transwell and incubated for 12 hours, followed by fixation and staining with crystal violet.

Wound healing assay was used to assess cell motility in two dimensions. Tumor cells grown to monolayer were scratched by a pipette tip, followed by incubation with fresh medium for 12 hours. The reduction of the wound width relative to the wound width at time 0, when scratch was conducted, was expressed as % migration distance.

The assays were performed at 12 hours because the number of cells in miR-100 or anti-miR-100-transfected group is the same as that of their control groups at this time point.

### Luciferase reporter assay

QGY-7703 cells in a 48-well plate were co-transfected with 20 nM of miR-100 or NC duplex, 10 ng of psiCHECK2 luciferase reporter plasmids that contained either wild-type or mutant 3′-UTR of the target genes. Forty-eight hours after transfection, cell lysates were applied to luciferase assay as described [37].

### Analysis of gene expression

Gene expression was analyzed by semi-quantitative reverse-transcriptase polymerase chain reaction (RT-PCR), quantitative real-time RT-PCR (qPCR) or Western blotting.

Total RNA extraction and RT-PCR analysis were performed as described [37]. qPCR analysis for miR-100 expression was performed on a LightCycler 480 (Roche Diagnostics, Mannheim, Germany) using a TaqMan MicroRNA Assay kit (Applied Biosystems, CA, USA). All reactions were run in triplicate. The cycle threshold (Ct) values did not differ by more than 0.5 among the triplicates. The miR-100 level was normalized to RNU6B to permit the calculation of 2^−ΔΔCt^ value.

For Western blotting, proteins were separated in a 10% polyacrylamide gel and transferred to a methanol-activated PVDF membrane (Millipore, MA, USA). The membrane was blocked for 2 hours in Tris-buffered saline-Tween-20 (TBST) containing 2% bovine serum albumin, and then immunoblotted subsequently with the primary and secondary antibodies. The protein level was detected using a luminal reagent (Millipore). The antibodies used for Western blotting included: mouse monoclonal antibodies against Rac1 (cat. 05-389, Millipore), β-actin (cat. BM0627, Boster, Wuhan, China), Akt (cat. 2967) or phospho-ser473-Akt (cat. 4051, Cell Signaling Technology, CST, MA, USA); rabbit polyclonal antibody against ICMT (cat. 51001-2-AP, Proteintech, IL, USA); horse anti-mouse (CST) or goat anti-rabbit (CST) HRP-conjugated secondary antibody.

### Immunohistochemical (IHC) staining

Formalin-fixed, paraffin-embedded tissues were cut into 4-μm sections, deparaffinized with xylene, rehydrated through graded ethanol, followed by quenching of endogenous peroxidase activity in 0.3% hydrogen peroxide and antigen retrieval by microwave heating in 10 mM citrate buffer (pH 6.0). Sections were incubated at 4 °C overnight with mouse monoclonal antibody against Rac1 or rabbit polyclonal antibody against ICMT, then immunostained using ChemMate DAKO EnVision Detection Kit, Peroxidase/DAB, Rabbit/Mouse (code K 5007, DakoCytomation, Glostrup, Denmark), which resulted in a brown-colored precipitate at the antigen site. Subsequently, sections were counterstained with hematoxylin (Zymed Laboratories, CA, USA) and mounted in non-aqueous mounting medium. All runs included a no primary antibody control.

Rac1 and ICMT expression were evaluated under a light microscope at a magnification of 400×. For each case, five representative areas were evaluated. For mouse xenografts, a total of 1000 to 2000 tumor cells were counted, and the proportion of positive staining cells was presented. For human samples, the intensity score was defined as no staining (0), weak (1), or strong (2) staining. The fraction score was then calculated based on the proportion of Rac1-stained cells (0%-100%). The intensity and fraction scores were multiplied to obtain a score, which ranged from 0 to 2 and represented the level of Rac1.

### F-actin staining for lamellipodia

Hepa-Ctrl and Hepa-miR-100 cells or QGY-7703 and SMMC-7721 cells that were transfected for 48 hours, were added to coverslip that had been pre-coated with 80 μg Matrigel (R&D Systems) in a 48-well plate, allowed to spread for 3 hours at 37 °C and then fixed, permeabilized and stained with fluorescent phalloidin (Life Technologies), a probe for filamentous actin.

### Detection of MMP2 activity in tumor cell-conditioned medium (TCM) by gelatin zymography

Forty-eight hours after transfection, tumor cells were washed with 1× PBS and then incubated in serum-free medium for 24 hours. TCM was collected and applied to acrylamide gel containing gelatin as described previously [37]. TCM loading was adjusted according to the number of live cells in each sample.

### Statistical analysis

The recurrence-free survival was calculated from the date of tumor resection to the time of first recurrence. Patients who were lost to follow-up or died from causes unrelated to HCC were treated as censored events, and patients who had received post-operative anticancer therapies prior to relapse were excluded from recurrence-free survival analysis. Kaplan-Meier survival curves were constructed and the differences between groups were analyzed using a log rank test with SPSS version 16.0 (SPSS Inc., Chicago, IL). Association between the miR-100 level and clinical characteristics of patients was analyzed by using unpaired Student *t* test.

Data were expressed as the mean ± standard error of the mean (SEM) from at least three independent experiments. The differences between the groups were analyzed by Student *t* test when two groups were compared or by one-way analysis of variance when more than two groups were compared. Analyses were performed with GraphPad Prism (version 5, GraphPad Software, Inc., San Diego, CA). Correlations between two variables were explored with the Spearman's correlation coefficient. All statistical tests were two-sided; *P* < 0.05 was considered statistically significant.

## SUPPLEMENTARY MATERIAL FIGURES AND TABLES


