# Supplementary material for: Downregulation of microRNA-100 enhances the ICMT-Rac1 signaling and promotes metastasis of hepatocellular carcinoma cells
Source: Oncotarget. 2014 Oct 18;5(23):12177–88. doi: 10.18632/oncotarget.2601 (PMC4322964; doi:10.18632/oncotarget.2601)
Supplement: Supplementary file 1 [file oncotarget-05-12177-s001.pdf]

Downregulation of microRNA-100 enhances the ICMT-Rac1 signaling and promotes metastasis of hepatocellular carcinoma cells

Supplementary Material

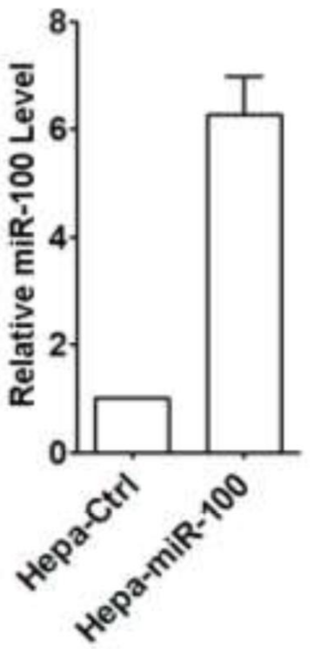

Supplementary Figure 1: Analysis of miR-100 expression in Hepa1-6 subclones. Hepa1-6 cells that stably expressed control vector (Hepa-Ctrl) or miR-100 (Hepa-miR-100) were analyzed by quantitative real-time PCR (qPCR). RNU6B was used as an internal control.

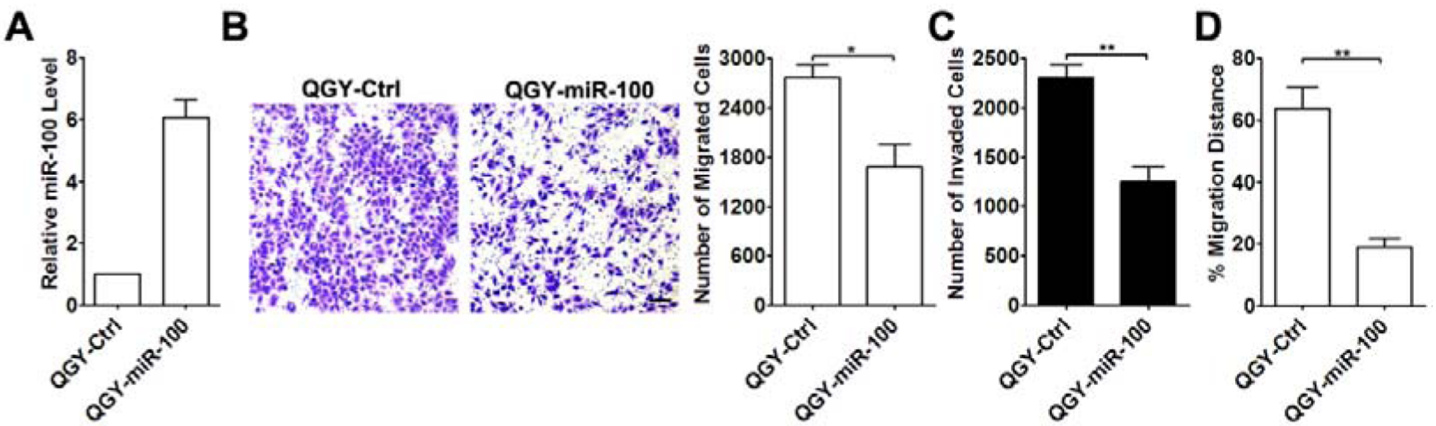

Supplementary Figure 2: Stable expression of miR-100 suppresses the *in vitro* migration and invasion of human HCC cells. (A) Analysis of miR-100 expression in QGY-7703 subclones. QGY-7703 cells that stably expressed control vector (QGY-Ctrl) or miR-100 (QGY-miR-100) were analyzed by qPCR. RNU6B was used as an internal control. (B and C) Restoration of miR-100 expression suppressed the *in vitro* migration and invasion of QGY-7703 cells. QGY-Ctrl and QGY-miR-100 were added to transwell chamber without (B, 3.5×10^4) or with (C, 7.5×10^4) Matrigel coating, then incubated for 12 hours, followed by staining with crystal violet. Scale bar, 50 μm. *P < 0.05, **P < 0.01. (D) Reintroduction of miR-100 repressed QGY-7703 mobility. QGY-Ctrl and QGY-miR-100 were applied to wound-healing assay for 12 hours. **P < 0.01.

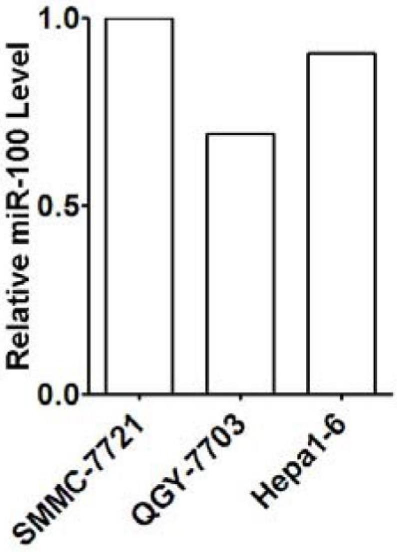

Supplementary Figure 3: Analysis of miR-100 expression in different hepatoma cell lines. Expression of mature miR-100 was detected by qPCR in SMMC-7721, QGY-7703 and Hepa1-6 cells. RNU6B was used as an internal control.

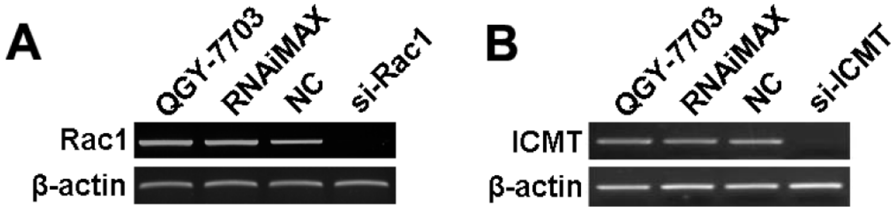

Supplementary Figure 4: Silencing of Rac1 and ICMT by siRNA. (A) Knockdown of Rac1 by si-Rac1. (B) Knockdown of ICMT by si-ICMT. QGY-7703 cells without treatment (lane 1), treated with Lipofectamine RNAiMAX (lane 2), or transfected with the indicated RNA duplex (lanes 3-4) were applied to RT-PCR analysis. β-actin, internal control.

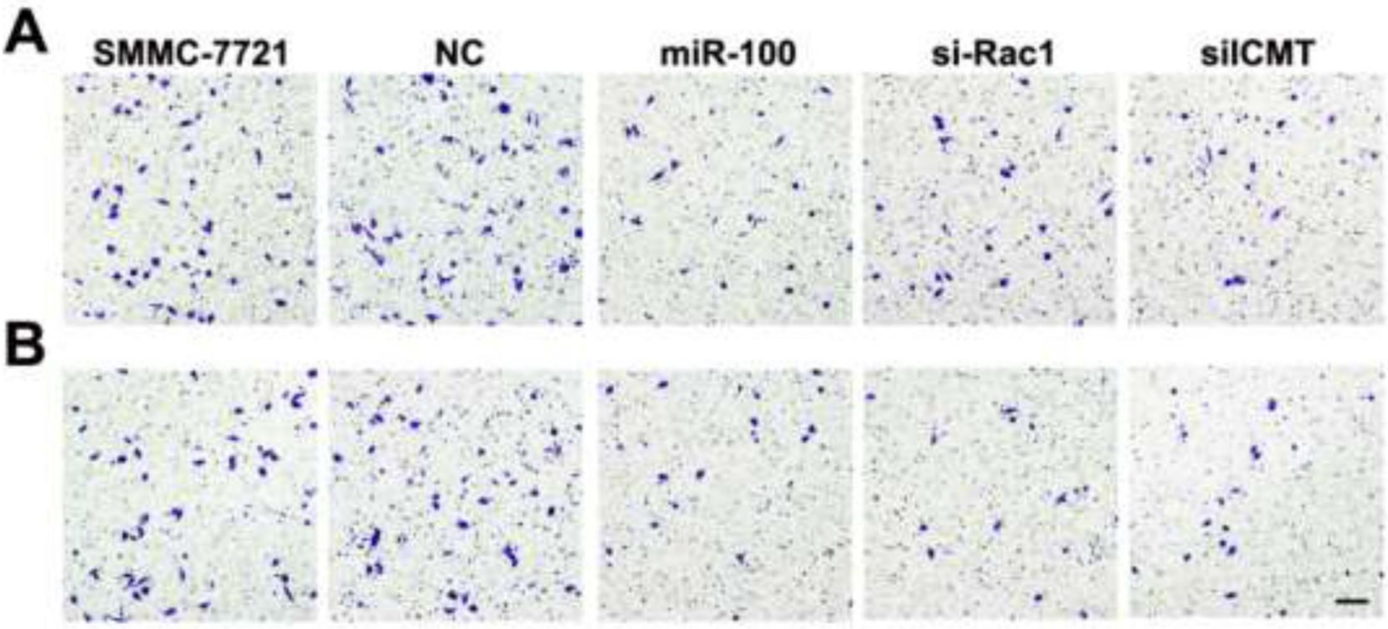

Supplementary Figure 5: miR-100 exerts its anti-metastasis function by suppressing the ICMT-Rac1 signaling. (A) Knockdown of either Rac1 or ICMT inhibited the migration of SMMC-7721 cells. (B) Knockdown of either Rac1 or ICMT inhibited the invasion of SMMC-7721 cells. SMMC-7721 cells transfected with indicated duplex were added to transwell chamber without (A, 5×10^4) or with (B, 7.5×10^4) Matrigel coating, then incubated for 12 hours, followed by staining with crystal violet. Scale bar, 50μm.

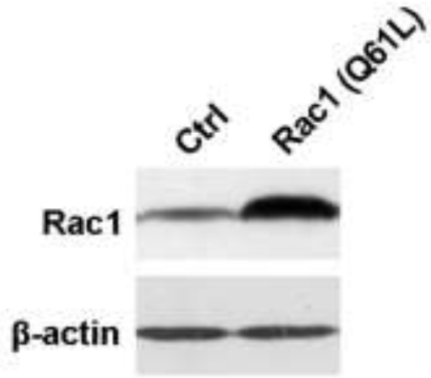

Supplementary Figure 6: Overexpression of the constitutively active Rac1. Forty-eight hours after transfection with control or Rac1 (Q61L) expression vector, QGY-7703 cells were analyzed by immunoblotting. β-actin, internal control.

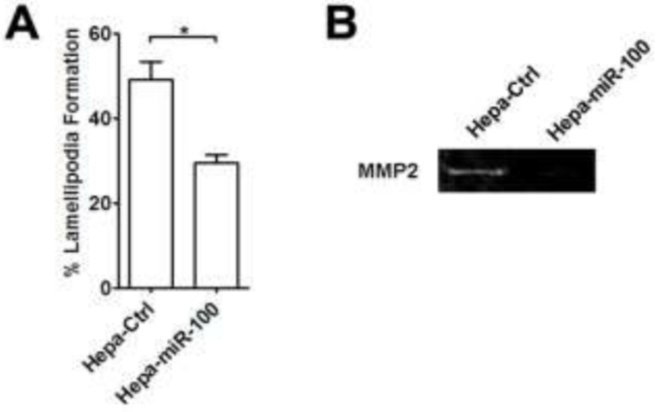

Supplementary Figure 7: miR-100 negatively regulates lamellipodia formation and MMP2 activity in Hepa1-6 cells. (A) Expression of miR-100 abrogated lamellipodia formation. Hepa-Ctrl and Hepa-miR-100 cells were seeded on Matrigel-coated plates for 3 hours, then fixed and stained with phalloidin to visualize F-actin. *$P < 0.05$. (B) Expression of miR-100 attenuated MMP2 activity. After cell seeding, Hepa-Ctrl and Hepa-miR-100 cells were refreshed with serum-free medium for 24 hours. TCM was then collected and subjected to gelatin zymography analysis. The results were reproducible in three independent experiments and the representative image is shown.

Supplementary Table 1. The predicted scores of miR-100 targets

	Context Score[a]	mirSVR score[b]
**Rac1**	-0.13	-0.1051
**ICMT**	-0.09	-0.3767

[a] The context score for a specific site was provided by TargetScan (Release 4.2). It is the sum of the contribution of these four features: site-type contribution, 3' pairing contribution, local AU contribution, position contribution.

[b] mirSVR, scored for likelihood of mRNA downregulation, was provided by MiRanda databases (August 2010 release). It is a regression model that is trained on sequence and contextual features of the predicted miRNA::mRNA duplex.

Supplementary Table 2. Sequences of DNA and RNA Oligonucleotides

Name	Sense Strand/Sense Primer (5' - 3')	Antisense Strand/Antisense Primer (5' - 3')
**miRNA and siRNA Duplexes**		
miR-100	AACCCGUAGAUCCGAACUUGUG	CAAGUUCGGAUCUACGGGUAAU
Si-Rac1	GGAGAUUGGUGCUGUAAAAdTdT	UUUUACAGCACCAAUCUCCdTdT
Si-ICMT	GAAUCACAGCCUGGAGUAUdTdT	AUACUCCAGGCUGUGAUUCdTdT
NC	UUCUCCGAACGUGUCACGUdTdT	ACGUGACACGUUCGGAGAAdTdT
**miRNA Inhibitors**		
Anti-miR-100	CACAAGUUCGGAUCUACGGGUU	
Anti-miR-NC	CAGUACUUUUGUGUAGUACAA	
**Primers for Gene and 3'-UTR Cloning**		
miR-100	TTAGAATTCAGTGGGACGAAGTCCTTTCC	ATTGGATCCGGAATTGAGGGCCAGCCTAT
Rac1 3'UTR	AGTGGGCCCGCGTAGCAGCTCAGCTCTTT	AGTTCTAGACTGCTCAGTCAGAAGGGGTTC
ICMT 3'UTR	ATGCTCGAGGATGTGGGAGCGACATCAAT	ATTGCGGCCGCCTCCCAAATTGCTGGGATTA
